# Feet and Shoes

**Published:** 1883-04

**Authors:** 


### FEET AND SHOES.

SWISS examining surgeons are compelled to reject, every year, 800 recruits—the strength of a battalion—for malformation of the feet resulting from badly-fitting shoes. The foot is, in reality, a bow so elastic that at every step it contracts and expands, lengthens and shortens, and a line drawn through the centre of the great toe intersects the heel. But shoemakers, who are generally utterly ignorant of the anatomy of the foot, do not give room enough for the lateral extension of the great toe. They crib, cabin and confine it until it is forced against the other toes. Hence arise frequent inflammations of the great toe—corns, ulcerations, and sometimes veritable articular inflammation. Another evil of bad shoeing is flat-footedness, whereby the arch is converted into a straight line, and prolonged walking and marching rendered impossible. Another cause of this effect is the habit of carrying heavy weights at an early age; but in most instances perfect shoes would restore the foot to its normal condition. The first obstacle to a reform in the shape of shoes lies in the fact that it would involve a great expense in the shape of new lasts—an expense that shoemakers are naturally loath to incur. Fashion has also its lasts, and shoemakers consider themselves bound to conform to the prevailing mode. A test of a perfect pair of shoes is that, when placed together, they would touch only at the toes and the heels; the soles should follow the sinuosities of the feet, and to give room for their expansion should exceed them in length by fifteen to twenty millimètres.

